# Different Neutralizing Antibody Responses of Heterologous Sera on Sheeppox and Lumpy Skin Disease Viruses

**DOI:** 10.3390/v16071127

**Published:** 2024-07-14

**Authors:** Francisco J. Berguido, Richard Thiga Kangethe, Wendy Shell, Viskam Wijewardana, Reingard Grabherr, Giovanni Cattoli, Charles Euloge Lamien

**Affiliations:** 1 Animal Production and Health Laboratory, Joint FAO/IAEA Centre, Department of Nuclear Sciences and Applications, International Atomic Energy Agency, Wagramer Strasse 5, P.O. Box 100, 1400 Vienna, Austriac.lamien@iaea.org (C.E.L.); 2 Institute of Biotechnology, University of Natural Resources and Life Sciences (BOKU), Muthgasse 18, 1190 Vienna, Austria; 3 Institute for Veterinary Disease Control, AGES—Austrian Agency for Health and Food Safety, Robert Koch-Gasse 17, 2340 Mödling, Austria

**Keywords:** capripoxvirus, LSDV, SPPV, GTPV, VNT, vaccine efficacy

## Abstract

Sheeppox virus (SPPV), goatpox virus (GTPV), and lumpy skin disease virus (LSDV) are the three members of the genus *Capripoxvirus* within the *Poxviridae* family and are the etiologic agents of sheeppox (SPP), goatpox (GTP), and lumpy skin disease (LSD), respectively. LSD, GTP, and SPP are endemic in Africa and Asia, causing severe disease outbreaks with significant economic losses in livestock. Incursions of SPP and LSD have occurred in Europe. Vaccination with live attenuated homologous and heterologous viruses are routinely implemented to control these diseases. Using the gold standard virus neutralization test, we studied the ability of homologous and heterologous sera to neutralize the SPPV and LSDV. We found that LSD and SPP sera effectively neutralize their homologous viruses, and GTP sera can neutralize SPPV. However, while LSD sera effectively neutralizes SPPV, SPP and GTP sera cannot neutralize the LSDV to the same extent. We discuss the implications of these observations in disease assay methodology and heterologous vaccine efficacy.

## 1. Introduction

Lumpy skin disease (LSD), sheeppox (SPP), and goatpox (GTP) are highly contagious viral diseases causing severe economic losses in the domestic ruminant industry [1]. Lumpy skin disease virus (LSDV), goatpox virus (GTPV), and sheeppox virus (SPPV), the causative agents of LSD, GTP, and SPP, respectively, are the three members of the genus *Capripoxvirus* within the family *Poxviridae* [2].

Within domestic ruminants, the SPPV and GTPV can infect both sheep and goats, while the LSDV infects cattle, water buffalo, and Yak [3,4]. Lumpy skin disease, sheeppox, and goatpox are listed as notifiable diseases by the World Organization for Animal Health (WOAH) [5].

The LSDV, GTPV, and SPPV have high sequence similarity (96–97% nucleotide identity) [2,6] and are serologically indistinguishable [7,8,9]. This is partly why, in the past, immunization with live attenuated versions of one of these viruses was believed to cross-protect against all others (universal vaccine) [10]. However, there is growing evidence that cross-protection is limited or nonexistent [11].

The gold standard for detecting antibodies against these diseases is the virus neutralization test (VNT) [12]. WOAH recommends its use as a diagnostic tool [13].

Several reports have shown the lack of effective cross-neutralization of virus variants, such as for the earlier and recent coronavirus studies [14,15]. For poxviruses, cross-neutralization varies within members of the genus [11,16,17].

During the characterization of sera for validation studies to develop capripox serological tests, we observed differences in the ability of heterologous sera to neutralize LSDV and SPPV.

The present study uses VNT to test SPP-, GTP-, and LSD-positive and -negative sera on the SPPV and LSDV to determine their neutralizing effect on each virus type. The results are relevant for establishing sound VNT methodologies and can contribute to elucidate heterologous vaccine efficacy for capripox.

## 2. Materials and Methods

### 2.1. Viruses

Wildtype LSDV Massalamia P4 isolate (Sudan) and Sheeppox virus Djelfa (Algeria), used in this study, were obtained from the Central Veterinary Research Laboratories, Animal Resources Research Corporation, Ministry of Livestock, Fisheries and Ranges, Khartoum, Sudan and the Institut National de la Médecine Vétérinaire, Laboratoire Central Vétérinaire d’Alger, Algiers, Algeria, respectively. Both viruses were propagated on an embryonic skin cell line from sheep (ESH-L cells) in Hank’s Minimum Essential Medium (MEM) supplemented with 10% fetal calf serum (FCS) and 1% antibiotics [18].

### 2.2. Virus Titration

Fifteen mL of ESH-L cell suspension containing 10^5^ cells/mL in DMEM without FCS was prepared, and 100 µL/well (10^4^ cells) was added in all wells of a 96-well plate. A serial ten-fold dilution, 2 mL/dilution, was prepared for each virus stock to be titrated in DMEM without FCS, starting from 10^−2^ and reaching 10^−6^. For each titration plate, the ten central wells of the same row were infected with 100 µL/well of each viral dilution. Ten control wells were also prepared with 100 µL of growth medium without FCS nor virus. The plates were incubated at 37 °C and observed daily for cytopathic effects (CPEs). The number of infected wells were counted, and the proportion of infected wells for each dilution and the titer between day 9 and 10 post-infection was calculated. LSDV and SPPV titers (TCID_50_) were calculated using the Kärber method [19]. The viral suspension was stored at −80 °C until use. All procedures were performed in the BSL-3 laboratory facilities at AGES, Austria.

### 2.3. Virus Neutralization Test

Briefly, in quadruplicate, heat-decomplemented (56 °C for one hour) serum samples and positive and negative controls were diluted, starting at 1/16 in DMEM cell culture medium (Thermo Fisher Scientific, Waltham, MA, USA), and were incubated with and without 100 TCID_50_ final concentration of wildtype LSDV Massalamia [20] or SPPV Djelfa (Algeria) for one hour at room temperature. Then, 20,000 ESH-L cells/well were added. The plates were incubated at 37 °C for eight days and then examined for CPEs. Serum samples were considered negative when CPEs were observed in at least two of four wells and positive when CPEs were blocked (protected) in at least three.

### 2.4. Sera Description

There were four types of reference population sera used for this study: (a) Naturally LSDV-infected cattle (*n* = 26) from the Republic of North Macedonia, kindly provided by Kiril Krstevski and Igor Djadjovski (School of Veterinary Medicine, University “Ss Cyril and Methodius”, Skopje, Republic of North Macedonia). These samples were confirmed positive by A34 [21] and IDVet (Grabels, France) ELISAs. (b) Sheeppox virus and goatpox virus experimentally infected, positive sheep sera (*n* = 3) from Laboratoire Central Vétérinaire (LCV) (Mali) [22] and goat sera (*n* = 4) from the Animal Health Institute (AHI) (Ethiopia) [23]. These samples were confirmed positive by A34 and IDVet ELISAs. (c) Bovine LSD-negative sera (*n* = 2) and (d) Capripox-negative goat (*n* = 5) and sheep (*n* = 2) sera from Austria (provided by AGES, Austria). Sera from (c) and (d) were obtained in countries where the disease is historically not present.

### 2.5. Data Analysis and Presentation

A one-tailed two-sample *t*-test was performed in R base, and graphical representations were carried out using ggplot 2 in R [24] to compare the differences between groups. The significance level was set at *p*-value (*p* < 0.1 ^+^, *p* < 0.05 *, *p* < 0.001 **, *p* < 0.0001 ***).

### 2.6. Antigenic Relatedness

The antigenic relatedness (R) between LSDV and SPPV was calculated by the Archetti and Horsfall Method (1951), expressed by the formula: R = (rA × rB)^^^1/2, where rA is the ratio of the heterologous titer obtained with virus B to the homologous titer obtained with virus A [25]. The value rB is the ratio of the heterologous titer obtained with virus A to the homologous titer obtained with virus B. A homologous R-value = 1. R values close to 1 show high antigenic similarity between two viral strains, R values between 0.70 and 0.33 indicate antigenic relatedness, and R values between 0.32 and 0.11 indicate loose relatedness. In contrast, R values below 0.11 indicate non-relatedness (serotype difference) [26,27,28,29,30].

## 3. Results

### 3.1. Fixed Sera Dilution VNT Using Heterologous and Homologous SPP, GTP, and LSD Sera

**Figure 1:**
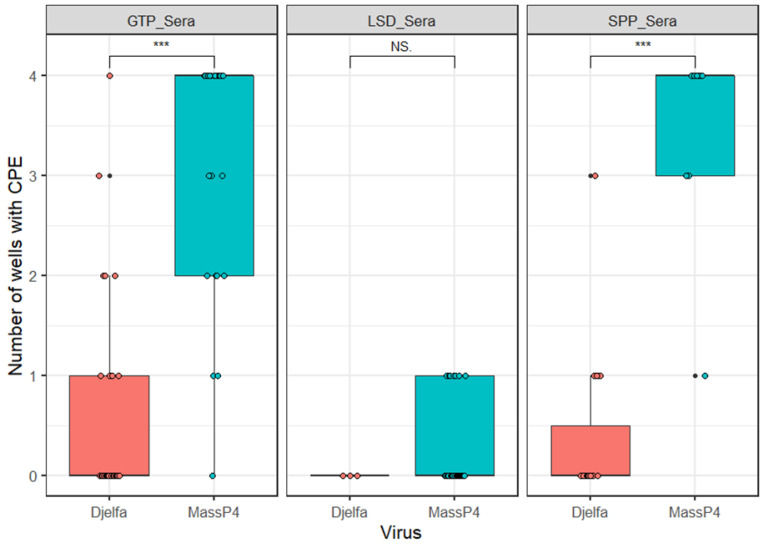
Results of VNT using homologous and heterologous sera on SPPV (Djelfa) and LSDV (Massalamia), as indicated by the number of individual wells with CPEs. GTP- and SPP-positive sera exhibited statistically significant neutralizing differences against SPPV and LSDV. *** shows one-tailed *t*-test significance values of = *p* < 0.0001. NS = not significant. The VNT was carried out at a fixed sera dilution of 1/16 in cell culture medium. Vertical lines indicate standard deviation.

SPP-, GTP-, and LSD-positive sera diluted at 1/16 were used to neutralize the SPPV (Djelfa) and LSDV (Massalamia), with the number of wells exhibiting CPEs used as an indicator. As can be seen in Figure 1, LSD-, GTP-, and SPP-positive sera effectively neutralized the SPPV. LSD-positive sera could effectively neutralize LSDV. However, the SPP and GTP sera did not effectively neutralize the LSDV (Massalamia).

### 3.2. LSDV VNT

**Figure 2:**
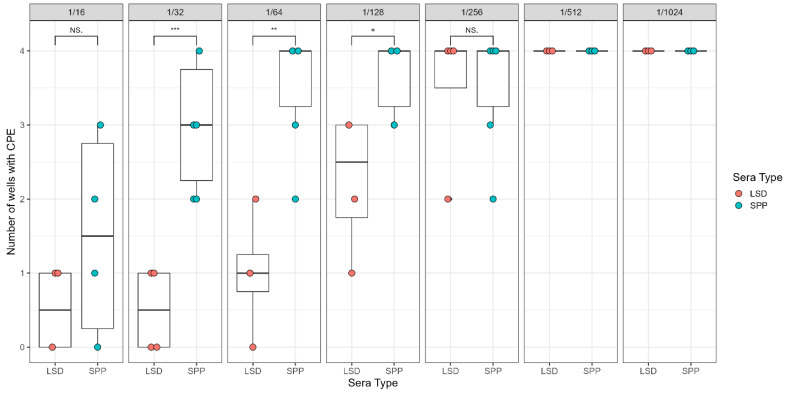
Results of LSDV VNT on homologous and heterologous sera neutralization using LSDV (Massalamia), as indicated by the number of individual wells with CPEs. LSD- and SPP-positive sera were serially diluted from 1/16 to 1/1024. Data for LSD- and SPP-positive sera were collected based on two independent experiments. “***”, “**”, and “+” represent one-tailed *t*-test significance values of *p* < 0.0001, 0.05, and 0.1, respectively. NS = not significant. Vertical lines indicate standard deviation.

Results from the LSDV VNT with sera dilutions 1/16 to 1/1024 show a dilution-dependent neutralizing effect with LSD sera dilutions up to 1/64. SPP sera at 1/16 were unable to effectively neutralize the heterologous virus (Figure 2).

### 3.3. SPPV VNT

**Figure 3:**
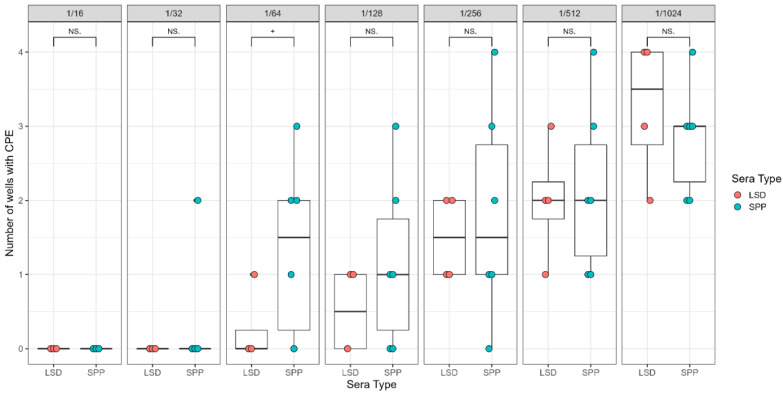
Results of SPPV VNT on homologous and heterologous sera neutralization using SPPV (Djelfa), as indicated by the number of individual wells with CPEs. LSD- and SPP-positive sera were serially diluted from 1/16 to 1/1024. Data for LSD and SPP positive sera were collected based on two independent experiments. “+” represents one-tailed *t*-test significance values of *p* < 0.1. NS = not significant. Vertical lines indicate standard deviation.

Results from the SPPV VNT with sera dilutions from 1/16 to 1/1024 show a dilution-dependent neutralizing effect with LSD sera diluted up to 1/256. SPP sera at 1/64 were unable to effectively neutralize the homologous virus (Figure 3).

### 3.4. Antigenic Relatedness

As seen in Table 1, the values obtained using the Archetti–Horsfall formula to determine antigenic relatedness among homologous and heterologous sera as well as viruses show some antigenic identity between the LSDV and SPPV (R = 0.4). VNT values and calculations to obtain R are shown in Supplementary Tables S1 and S2, respectively.

## 4. Discussion

To confirm the positive or negative status of the available SPP, GTP, and LSD sera, we tested them using a singly diluted (1/16) VNT. The test, which used a titrated virus, was designed to rapidly determine the status of the sera. We observed differences in the results when SPP-positive sera were tested with the heterologous versus homologous virus. Furthermore, we tested serial dilutions of positive and negative SPP and LSD sera on the LSDV and SPPV to determine the effective virus neutralization of the serum titers and calculate an average.

The serological gold standard for detecting antibodies against capripoxvirus diseases is the virus neutralization test (VNT) [12]. The SPPV, GTPV, and LSDV have high sequence similarity (96–97% nucleotide identity) [2,6] and are serologically indistinguishable [7,8,9]. Additionally, cross-immunity has been reported [31]. Under this principle, the WOAH Manual of Diagnostic Tests and Vaccines for Terrestrial Animals [13] suggests, in the example of the sheeppox and goatpox VNT protocols, the use of SPP and GTP sera on LSDV.

In this study, we found that, by VNT, while LSD sera effectively neutralizes both the LSDV and SPPV, SPP sera effectively neutralize the SPPV but does not neutralize the LSDV in the same proportion. Additionally, the first set of results suggests that although not statistically significant, there was a better neutralizing effect (higher dilution) of GTP sera than SPP sera on LSDV.

Although we only perform the VNT using one cell type, and additional testing is required for validation, the present study’s results agree with the studies advocating against heterologous viral use [11]. As a minimum, this study indicates the need to update the current examples in the WOAH Manual of Diagnostic Tests and Vaccines for Terrestrial Animals by advocating against the use of capripox sera on heterologous viruses in VNT, particularly for SPP. The current VNT example of the WOAH Manual of Diagnostic Tests and Vaccines for Terrestrial Animals, under sheeppox and goatpox, uses SPP or GTP sera on the KSGP O-240 vaccine strain, which is an LSD virus [13].

Our results also align with multiple reports of incomplete protection and failed vaccinations associated with heterologous vaccinations, especially concerning the use of SPPV-attenuated vaccines against LSD. Cross-protection of LSD heterologous vaccines is, at best, only partial [12,32], with higher doses of the SPPV and GTPV vaccine strains usually being applied to increase the efficacy against LSD.

Sheeppox vaccines used for cattle immunization against LSD, even at doses 10 times higher than for small ruminants, showed only partial cross-protection, which could result in virus propagation and potential spread [32,33,34].

The Bakirköy SPPV vaccine at four times the sheep dose has been used in Turkey against LSD. The RM65 SPPV vaccine at ten times the sheep dose has been used in cattle across the Middle East. The Romanian SPPV vaccines have been used in cattle in Egypt [34,35,36,37].

Although immunity against capripoxviruses is predominantly cell-mediated, an initial robust humoral response is essential to neutralize the viruses [38].

For LSD, in many instances, heterologous vaccines are a cheaper and faster alternative. Some reports suggest that the attenuation of the goatpox virus and sheeppox virus can be easier than the lengthy and costly attenuation of the LSDV [39,40,41]. However, before their use, an adequate evaluation and determination of their vaccines’ efficacy must be established [12].

Additionally, we used the Archetti–Horsfall formula, which shows the relatedness of antigens to antibodies in homologous and heterologous sera via indexes. Although, as shown by the multiple references, there is no universal threshold for this formula; however, many publications refer to R values close to 1 as showing a high antigenic similarity between two viral strains. R values between 0.70 and 0.33 are described as showing antigenic relatedness; R values between 0.32 and 0.11 indicate loose relatedness. In the same fashion, R values below 0.11 indicate non-relatedness (serotype difference). The antigenic relatedness value obtained between the LSDV and SPPV was 0.4. This value falls within the antigenic relatedness range but approaches values close to loose relatedness. This result suggests that although the two viruses cannot be considered as producing distinct serotypes, they show enough antigenic diversity to provide an additional explanation to the results presented here.

A limitation of this study is that we did not have the GTPV available to include it in the final assessment. Our future studies will address this limitation.

Our results indicate different antibody responses from the two infectious capripoxvirus genus members and suggest that using homologous sera in capripox VNT protocols is more reliable. Our study also partially supports reports of incomplete protection being associated with heterologous vaccinations, as humoral protection plays a role in the early stages of capripoxvirus infections.

## Figures and Tables

**Table 1:** Antigenic relatedness between SPPV and LSDV. Archetti–Horsfall formula was used to determine the antigenic relatedness between viral strains in a cross-virus neutralization test.

	Antiserum	
Virus	SPP	LSD
SPPV	1	0.4
LSDV	-	1

## Data Availability

The data presented in this study are available upon request from the corresponding author.

## Supplementary Materials

The following supporting information can be downloaded at: https://www.mdpi.com/article/10.3390/v16071127/s1, Table S1: Average values of SPP and LSD homologous and heterologous VNT; Table S2: R-value calculation.
